# Development of the Taiwanese version of the *Health Enhancement Lifestyle Profile (HELP-T)*

**DOI:** 10.1371/journal.pone.0199255

**Published:** 2018-06-26

**Authors:** Fiona Pei-Chi Su, Ling-Hui Chang, Hui-Fen Mao, Eric J. Hwang

**Affiliations:** 1 School of Occupational Therapy, College of Medicine, National Taiwan University, Taipei, Taiwan; 2 Department of Occupational Therapy and; 3 Institute of Allied Health Sciences, College of Medicine, National Cheng Kung University, Tainan, Taiwan; 4 Department of Physical Medicine and Rehabilitation, National Taiwan University Hospital, Taipei, Taiwan; 5 Department of Occupational Therapy, College of Health, Human Services and Nursing, California State University, Dominguez Hills, Carson, CA, United States of America; National University of Singapore, SINGAPORE

## Abstract

**Objectives:**

To develop and validate a Taiwanese version of the *Health Enhancement Lifestyle Profile* (*HELP-T*) for community-dwelling older Taiwanese adults (≥ 55 years).

**Methods:**

The original *Health Enhancement Lifestyle Profile* (*HELP*) is a 56-item self-report questionnaire measuring various aspects of health-related lifestyles in older adults. The standard cultural-adaptation procedure was used for questionnaire translation and modification. A field test was conducted for culturally specific item selection, rating-scale analysis, and psychometric validation of the *HELP-T* in a sample of 274 community-dwelling older adults via classical test theory.

**Results:**

The 59-item *HELP-T* is culturally adapted from the original 56-item *HELP*. The original 6-point rating scale was modified to a 3-point scale for easy use by Taiwanese older adults. The *HELP-T* had good internal consistency (Cronbach’s alpha = 0.82). The test-retest reliability for the total score was high (0.92), and moderate to high (range: 0.57–0.92) for subscales. The construct validity was supported by the significant correlations between each subscale and the total score (Spearman’s *rho* = 0.41–0.67, *p* < 0.0001) and by the ability of the scores to significantly discriminate between participants with different levels of self-rated health (*p* = 0.0001).

**Conclusions:**

The *HELP-T* is a suitable clinical tool for assessing and monitoring lifestyle risk factors, establishing client-centered lifestyle intervention goals, and determining the outcomes of lifestyle interventions.

## Introduction

Taiwan is one of the world’s most rapidly aging nations. Between 1993 and 2018, the elderly population (≥ 65 years) almost doubled from 1.49 million (7% of the entire population) to 2.9 million (14%); this number is predicted to will reach approximately 4.4 million (20%) in 2026 [1]. National statistics for 2016 [2] showed mean life expectancy at 80.0 years but healthy life expectancy at only 71.0 years, which indicates an 8- to 9-year duration for healthcare services. On average, elderly Taiwanese have 2 or more chronic diseases, and 0.48 million of them are expected to require long-term care [3]. This will present a formidable challenge to families, healthcare providers, the government, and the entire community.

Early in 1980, Dr James Fries, the “healthy-aging pioneer”, hypothesized that active and healthy lifestyles would minimize the duration of chronic diseases, postpone the onset of disability and premature death, and decrease the amount of disability among all adults [4]. Therefore, a paradigm shift in aging care is needed to emphasize the strategies of disease prevention and promotion of healthy lifestyles [5].

The term “health-related lifestyle” comes from the idea that a person’s daily pattern of activities can be judged healthy or unhealthy. A healthy lifestyle is generally characterized as a “balanced life” in which one makes “wise choices” to engage in multidimensional daily activities to maintain or improve one’s health [6]. Habitual health-promoting behaviors—e.g., self-actualization, health responsibility, exercise, healthy diet, interpersonal support, and stress management—are considered the core of a healthy lifestyle [7].

Literature across disciplines [8–13], has also identified lifestyle as a modifiable factor and has integrated it into the framework of successful aging to promote health and prevent chronic illnesses among older adults. One study [14] reported that cumulative lifetime disability was four times greater in elderly people with unhealthy lifestyles such as smoking, unhealthy diet, and lack of exercise than in those with healthy lifestyles. Despite abundant evidence and published guidelines calling for healthy lifestyle interventions, there is a paucity of clinical assessments that enable health professionals to systematically assess and identify an older adult’s lifestyle risk factors, to monitor the change of specific behaviors, and to measure the outcomes of services. The *Health-Enhancement Lifestyle Profile* (*HELP*) was developed to fill this gap.

The *HELP* was designed for screening and monitoring health-related lifestyle factors and for examining the outcomes of interventions aimed at promoting healthy lifestyles for older adults. The *HELP* broadly defines lifestyle through the physiological, social, and spiritual dimensions of health [15]. It contains the following scales: (1) *Exercise*, (2) *Diet*, (3) *Work*, *Education*, *and Social Participation*, (4) *Leisure*, (5) *Activities of Daily Living*, (6) *Stress Management and Spiritual Participation*, and (7) *Other Health Promotion and Risk Behaviors*. The psychometric properties of the *HELP* were supported using a Rasch measurement model and classical test theory (CTT), with data derived from samples of community-dwelling older adults (≥ 55 years) who lived in southern California [15, 16].

One’s health-related lifestyle is not only one’s personal choice and responsibility; it is also influenced by environmental and cultural factors [17]. An instrument that measures lifestyle behaviors in one cultural group might not be appropriate for use in another cultural group. For example, differences in leisure and social activities might be found between older adults in Taiwan and those in the U.S. In addition, translating an assessment questionnaire from one language to another might cause misunderstandings because of literal and idiomatic differences between the two languages and cultures [18]. Therefore, for cross-cultural use, instruments such as the *HELP* should be adapted to the target society and culture. Moreover, the validity and reliability of the adapted instrument must be determined.

This study aimed to develop a Taiwanese version of the *HELP* (*HELP-T*). For proper cross-cultural use, standard procedures were adopted to linguistically and culturally adapt the *HELP*, and CTT was used to determine the appropriateness of the rating scales and to confirm the reliability and validity of the *HELP-T*.

## Methods

### Instrument

The *HELP* has two major sections: (1) personal and health information: demographics, diagnoses and self-rated health (i.e., excellent, good, fair, or poor), and (2) seven subscales aforementioned that measure different aspects of a health-related lifestyle. Each subscale contains eight items that ask how often a person engaged s in various health-promoting or risky behaviors during the previous 3 months; a 6-point rating scale is used: never (score 0), 1–3 days/month (score 1), 1–2 days/week (score 2), 3–4 days/week (score 3), 5–6 days/week (score 4), or 7 days/week (score 5). For each *HELP* subscale, a total score between 0 and 40 can be computed: a higher score means a higher frequency of health-promoting behavior [15].

### Study phases

The study was conducted in two phases: (1) generating the preliminary *HELP-T*, and (2) evaluating the appropriateness of the rating scale and determining the intrument's reliability and validity. National Taiwan University Hospital’s Institutional Review Board approved the study (201203041RIC). Written informed consent was obtained from all participants.

#### Phase one: Generating the preliminary *HELP-T*

A series of procedures were adopted to generate a preliminary version of the *HELP-T*.

**Translating and culturally adapting the HELP**

The *HELP* was translated using a forward and backward translation procedure [18]. A review committee consisting of 6 experts including the research team (authors), 4 occupational therapists, one nurse, and one physical therapist, all of whom are experienced with geriatric care resolved wording discrepancies and determined conceptual and semantic equivalence between the two versions. Finally, the author of the original *HELP*, who was proficient in both languages, approved the two versions.

Several items from the original *HELP* were modified for cultural appropriateness. First, items with activities or objects with which older Taiwanese adults are usually not familiar were modified. For example, we replaced canned soup, hot dogs, bacon, sausage with local foods such as pickled cucumber, fermented bean curd, and kimchi in a *Diet* item. Second, some items were added with more activity examples that are culturally relevant; for example, “mahjong” was added to a *Leisure* item and Asian martial arts to an *Exercise* item. Moreover, because negatively worded questions are not commonly used in Mandarin, we rephrased them accordingly; for example, “How often during a week do you tend to ignore the routine for grooming and personal hygiene?” was revised to “How often during a week do you perform grooming and personal hygiene?”

**Creating additional culturally specific items**

We conducted three focus groups to gather information about health-related lifestyles from different perspectives. This procedure aimed to explore addtional culturally specific items that were not included in the original *HELP*. The first focus group included eight healthcare professionals (two occupational therapists, a physical therapist, a physician, a nurse, a dietician, a social worker, and a public health policy maker) specialized in geriatric care. The other two focus groups were separately conducted in southern and northern Taiwan, each with eight community-dwelling older adults of different ages and sex. Members of the focus groups discussed their definitions, experiences and perceptions regarding healthy and unhealthy activities of daily living. The same review committee analyzed the minutes of the focus groups and suggested eight culturally specific items (one for the *Exercise* subscale, four for the *Social and Productive* subscale, one for the *Leisure* subscale, and one original item [separated into two items] for the *Leisure* subscale) (see Results).

**Examining culturally specific items**

We used various criteria to determine the psychometric properties of the new items: (a) items with a mean between 1 and 4 (to prevent a floor or ceiling effect), (b) items with a median between 1 and 4, (c) correlation to the domain: *r* > 0.4, (d) a within-the-domain item-deleted reliability < 0.7, and (e) a significant difference in the mean scores between highest and lowest 1/3 groups [19]. The preliminary version of the *HELP-T* included the 56 original items and those culturally specific items that met the 5 criteria above (see Results).

#### Phase 2: Evaluating the appropriateness of the *HELP-T*’s rating scale, and of its reliability and validity

**Participants**

We enrolled 274 community-dwelling older adults (age > 55) who were cognitively intact and able to communicate in Mandarin or Taiwanese. Convenience and snowball sampling methods were used to recruit participants from a variety of diverse community sites across different regions of Taiwan.

**Data collection procedures**

The preliminary version of the *HELP-T* was administered through on-site paper-and-pen questionnaires in groups or face-to-face interviews by the first author. About 20 to 40 minutes were needed to complete the *HELP-T*.

**Data analysis**

Negatively conceptualized items were reverse coded for scoring. Kolmogorov-Smirnov tests and distribution plots were used to examine the normally hypothesis of the *HELP-T* total score and subscales. Therefore, parametric and non-parametric statistics were used in the following analyses, respectively.

**Examining and modifying the rating scale**

Many participants who completed the *HELP-T* through a face-to-face interview commented that the 6-point scale was too detailed and that they had difficulty in choosing their answers. Some response categories were rarely selected (< 10% of the participants). For more than half the items, participants used only 3 or fewer response categories. Therefore, we collapsed the 6-point scale into a 3-point scale by combining adjacent categories. As a result, a new 3-point rating scale was proposed: never or 1–3 days/month (score = 0), 1–4 days/week (score = 1), and 5–7 days/week (score = 2). Because the correlation coefficients between the scores of two rating scales was 0.985, we used the new 3-point rating scale for the subsequent analyses.

**Examining reliability and validity**

Cronbach’s alpha (α) was used to determine the internal consistency for the total score of the *HELP-T*. An α of at least 0.80 was considered good [20]. For test-retest reliability, 28 participants completed the *HELP-T* twice within an interval of 11–14 days. The intraclass correlation coefficient (ICC) with a one-way random effects model (1,1) [19, 21] was used to determine test-retest reliability of the *HELP-T* total score and subscale scores. An ICC of at least 0.75 was considered high, between 0.75 and 0.40 was considered moderate, and less than 0.40 was considered low [19].

The construct validity of the *HELP-T* was examined using hypothesis testing and discriminant validity. The hypothesis testing method evaluated the correlations of scores from the seven subscales and the total *HELP-T* score. We hypothesized that there would be significantly moderate-to-high correlations across the subscale scores and the total score, and that there would be small-to-moderate correlations across the seven subscales. Because the normality of the subscales was not assumed, Spearman’s correlation coefficient (*rho* [ρ]) was used for this hypothesis testing. Discriminant validity was used to test the *HELP-T* scores from participants who rated their health as excellent or good, and those who rated their health as fair or poor. An independent *t* test or Mann-Whitney U test (if normality was not assumed) was used to compare scores between the two groups.

SPSS 20 (IBM Corp., Somers, NY, USA) was used for all statistical analyses. Significance was set at *p* < 0.05. For multiple testing, significance was adjusted using the Bonferroni correction.

## Results

### Participant characteristics

Two hundred seventy-four older Taiwanese adults (mean age: 74.05 ± 9.85 years; 155 [56.6%] women) participated in this study (Table 1).

**Table 1:** Demographics and health-related data of participants (n = 274).

Characteristic	No. (%)
**Age** (years) (mean ± SD)	74.05 ± 9.85
56–64	60 (21.9)
65–74	97 (35.4)
75–84	64 (23.4)
85–97	49 (17.9)
No response	4 (1.5)
**Education**	
No formal education	12 (4.4)
Elementary school	48 (17.5)
Junior high school	25 (9.1)
Senior high school	59 (21.5)
Associate degree	44 (16.1)
Bachelor’s degree	64 (23.4)
Master’s degree and above	15 (5.5)
No response	6 (2.2)
**Marital status**	
Single	9 (3.3)
Married	199 (72.6)
Divorced	14 (5.1)
Separated	2 (0.7)
Widowed	48 (17.5)
No response	2 (0.7)
**Subjective health**	
Excellent	10 (3.6)
Good	74 (27.0)
Fair	171 (62.4)
Poor	16 (5.8)
No response	3 (1.1)
**Sex**	
Male	119 (43.4)
Female	155 (56.6)
**Employed**	
Full-time	35 (12.8)
Part-time	19 (6.9)
Unemployed or retired	219 (79.9)
No response	1 (0.4)
**Living status**	
Living alone	24 (8.8)
**No. of chronic diseases** (mean ± SD)	2.22 ± 1.92
0	36 (13.1)
1	87 (31.8)
2	56 (20.4)
3	37 (13.5)
4	17 (6.2)
5	21 (7.7)
6	8 (2.9)
7	6 (2.2)
8	6 (2.2)
**Religion**	
None	50 (18.2)
Buddhism	143 (52.2)
Daoism	33 (12.0)
Catholicism	4 (1.5)
Christian	36 (13.1)
I-Kuan Tao	3 (1.1)
Others	4 (1.5)
No response	1 (0.4)

SD: standard deviation.

### Selecting culturally specific items

Among the eight culturally specific items suggested, four met all criteria and were selected: “*gather with family members you don’t live with*”, and “*contact family members you don’t live with*” in the *Social and Productive Activities* subscale, and “*do art and music activities*, *play musical instruments*, *or sing (karaoke)*” and *“do gardening*, *planting*, *or crafts”* (split from one original item) in the *Leisure* subscale (Table 2). Therefore, the final version of the *HELP-T* includes 59 items: 10 in *Social and Productive Activities*, 9 in *Leisure*, and 8 in the other 5 subscales.

**Table 2:** Statistics for the culturally specific items for the preliminary *HELP-T* (6-point scale) and summary of item selection (n = 274).

Domain	Item	Mean	Median	*r* of	Item-del. rel.	Mean diff. (*p*)	Met *n*/5	Selected
	How many times per week do you:			domain	w/in domain	Highest 1/3-	criteria	Item?
						lowest 1/3		
Exercise	dance as an exercise?	0.66^#^	0^#^	0.40^#^	0.72^#^	0.89 (< 0.001)	1/5	No
Social	gather with family members you don’t live with?	1.34	1	0.59	0.63	1.03 (< 0.001)	5/5	Yes
Social	contact family members you don’t live with?	2.07	2	0.52	0.65	1.30 (< 0.001)	5/5	Yes
Social	take care of grandchildren?	1.34	0^#^	0.33^#^	0.71^#^	0.93 (< 0.001)	2/5	No
Social	go out for paid work?	0.66^#^	0^#^	0.17^#^	0.71^#^	‒0.04 (0.983) ^#^	0/5	No
Leisure	grow flowers or vegetables and fruit? *	1.59	1	0.48	0.50	1.01 (< 0.001)	5/5	Yes
Leisure	do crafts, art, or music activities (singing, playing instruments, drawing, handicrafts)?*	1.49	1	0.50	0.48	1.11 (< 0.001)	5/5	Yes
Leisure	cook as a hobby?	1.22	0^#^	0.40	0.52	1.11 (< 0.001)	4/5	No

Selection criteria: mean ≥ 1 or ≤ 4 (range 0–5); median = 1–4; *r* > 0.4 (non-negative or coefficient significantly correlated within [w/in] domain); Item-del. rel. (item-deleted reliability) decreased compared with domain reliability item-del. rel. w/in domain: *r* < 0.7); significant mean difference (diff.) between highest 1/3 and lowest 1/3. Items that met all criteria were selected.

^#^: did not meet all criteria

*: Split-form (1 original item).

### Descriptive data

The mean ± SD total score of the 59-item *HELP-T* (3-point rating scale) was 53.59 ± 11.41 (Table 3). We divided the mean of each subtotal score by the number of items in each subscale. The mean total scores of the *Activities of Daily Living* (1.45 ± 0.63), *Diet* (1.44 ± 0.67), and *Other Health Promotion and Risk Behaviors* (1.29 ± 0.67) subscales were right-skewed; thus, participants did them more than “*1–3 days/month*” but less than “*5–7 days/week*”. The *Exercise* (0.59 ± 0.67), *Leisure* (0.58 ± 0.64), and *Social* (0.41 ± 0.59) subscales had the lowest mean total scores. They were left-skewed; thus, participants did them less than “*1–4 days/week*”.

**Table 3:** Statistics for the 59 items of the 3-point *HELP-T* scale (n = 274).

Characteristic	Mean ± SD	Median	Cronbach’s	ICC for each	Corrected item-
			α	item or	total correlation
				subscale	(to subscale)
				(n = 28)	
**I. Exercise (range: 0–16)**	**4.74 (3.09)**	**4**	**0.71**	**0.92**	**-**
1. Walk for 20 min	1.27 (0.73)	1	0.68	0.63	0.40
2. Yoga or stretching exercises	1.08 (0.78)	1	0.65	0.79	0.52
3. Go to the gym or exercise at home	0.74 (0.80)	1	0.62	0.74	0.60
4. Perform strengthening exercises	0.38 (0.64)	0	0.65	0.85	0.53
5. Bike, jog, or hike	0.57 (0.73)	0	0.66	0.72	0.47
6. Swim, surf, etc.	0.13 (0.44)	0	0.73	1	0.06
7. Play sports	0.19 (0.52)	0	0.71	0^‡^	0.19
8. Perform martial arts (e.g., qi-gong)	0.38 (0.68)	0	0.69	0.73	0.32
**II. Diet (range: 0–16)**	**11.51 (2.66)**	**12**	**0.56**	**0.64**	**-**
1. Healthy foods rich in protein	1.37 (0.65)	1	0.53	0.36^‡^	0.28
2. Healthy foods rich in calcium	1.25 (0.68)	1	0.56	0.78	0.20
3. Three servings of fruits or vegetables	1.56 (0.63)	2	0.50	0.75	0.38
4. Three servings of whole-grain foods	1.23 (0.75)	1	0.56	0.54	0.19
5. Foods high in cholesterol	1.39 (0.68)	2	0.55	0.13^‡^	0.22
6. Foods high in sodium	1.49 (0.69)	2	0.50	0.44	0.37
7. Foods high in saturated/trans fat	1.59 (0.64)	2	0.52	0.28^‡^	0.31
8. Two servings of sweets or dessert	1.62 (0.62)	2	0.53	0.75	0.27
**III. Social and Productive Activities (range: 0–20)**	**4.06 (3.09)**	**4**	**0.71**	**0.86**	**-**
1. Go out with friends or relatives	0.60 (0.66)	1	0.68	0.47	0.43
2. Do volunteer work	0.32 (0.57)	0	0.70	0.84	0.30
3. Participate in a special activity or hobby group	0.57 (0.66)	0	0.68	0.91	0.43
4. Go to a senior citizen center	0.57 (0.68)	0	0.70	0.95	0.31
5. Participate in a social, cultural, or support group	0.28 (0.55)	0	0.67	0.75	0.47
6. Take part in political or community activity	0.10 (0.37)	0	0.69	-^#^	0.41
7. Participate in informal/non-academic classes	0.32 (0.55)	0	0.69	0.76	0.33
8. Go to a formal/academic class	0.10 (0.37)	0	0.69	‒0.05^‡^	0.43
9. Go to family gatherings^†^	0.43 (0.64)	0	0.69	0.62	0.37
10. Contact family members you don’t live with^†^	0.78 (0.71)	1	0.70	0.61	0.34
**IV. Leisure (range: 0–18)**	**5.23 (2.58)**	**5**	**0.50**	**0.75**	**-**
1. Read newspapers, magazines, etc.	1.22 (0.85)	1	0.45	0.64	0.26
2. Watch a favorite show on TV	1.62 (0.68)	2	0.51	0.48	0.08
3. Go out for sports, games, movies, etc.	0.33 (0.59)	0	0.44	0.35^‡^	0.31
4. Grow flowers or vegetables and fruit^†^	0.58 (0.77)	0	0.49	0.42	0.16
5. Play chess, bridge, cards, bingo	0.17 (0.47)	0	0.47	0.53	0.22
6. Write diaries, journals, short stories	0.28 (0.63)	0	0.46	0.96	0.24
7. Picnic, fish, sail, etc.	0.33 (0.58)	0	0.45	0.55	0.27
8. Do carpentry, auto-repair, or house-repair	0.13 (0.39)	0	0.47	0.37^‡^	0.21
9. Crafts, art, or music activities^†^	0.55 (0.72)	0	0.46	0.872	0.24
**V. Activities of Daily Living (range: 0–16)**	**11.62 (2.45)**	**12**	**0.53**	**0.71**	**-**
1. Do routine for hygiene	1.89 (0.36)	2	0.50	-^#^	0.31
2. Do routine for bathing	1.82 (0.43)	2	0.50	0.48	0.29
3. Stay up late at night	1.69 (0.59)	2	0.50	0.41	0.24
4. Go food or merchandise shopping	0.72 (0.68)	1	0.52	0.48	0.20
5. Skip one or more meals per day	1.76 (0.50)	2	0.55	0.18^‡^	0.08
6. Feel you don’t get enough rest	1.59 (0.63)	2	0.53	0.49	0.16
7. Do housework	1.15 (0.82)	1	0.45	0.31^‡^	0.36
8. Prepare or plan a meal	1.01 (0.88)	1	0.42	0.63	0.41
**VI. Stress management and spiritual participation (range: 0–16)**	**6.01 (3.24)**	**6**	**0.68**	**0.79**	**-**
1. Satisfied with your life	1.25 (0.74)	1	0.66	0.56	0.34
2. Do things that bring good moods	1.16 (0.75)	1	0.65	0.52	0.39
3. Talk with a special friend	0.92 (0.71)	1	0.66	0.66	0.32
4. Pray, worship, chant, etc.	0.65 (0.83)	0	0.66	0.84	0.34
5. Read spiritual/religious books	0.42 (0.69)	0	0.62	0.59	0.49
6. Go to church, temple, mosque, etc.	0.33 (0.57)	0	0.66	0.76	0.32
7. Watch spiritual/religious programs	0.51 (0.73)	0	0.64	0.74	0.41
8. Meditate, do yoga, or relax	0.78 (0.79)	1	0.65	0.66	0.36
**VII. Other health promotion and risk behaviors (range: 0–16)**	**10.34 (2.36)**	**2**	**0.40**	**0.57**	**-**
1. Drink three servings of alcohol in one day	1.93 (0.34)	2	0.43	0.12^‡^	‒0.07
2. How often do you smoke per month	1.88 (0.46)	2	0.40	-^#^	0.10
3. Take pain medicine	1.77 (0.58)	2	0.45	0^‡^	‒0.03
4. Take over-the-counter drugs	1.81 (0.55)	0	0.44	‒0.04^‡^	‒0.01
5. Read health-related articles	0.71 (0.84)	1	0.22	0.69	0.40
6. Watch health-related programs	0.98 (0.85)	1	0.27	0.67	0.33
7. Monitor your health at home	0.91 (0.90)	0	0.35	0.85	0.21
8. Attend health-promotion programs	0.34 (0.66)	10	0.30	0.79	0.32
**All 59 items (range: 0–118)**	**53.59(11.41)**	**53**	**0.82**	**0.92**	-

^†^Culturally specific items.

^‡^Spearman’s *r* for each item and the total score of its hypothesized domain does not reach the acceptable level of *r* > 0.4.

^#^The calculation of ICC failed because the scale or part of the scale of this item has zero variance.

### Reliability

The Cronbach’s α of the *HELP-T* total score was 0.82, which indicates good internal consistency. The test-retest reliability (ICC [95% CI]) of the *HELP-T* total scores was 0.92 (0.83–0.96), which indicated excellent score agreement. Four of the seven subscales (*Exercise*, *Social and Productive Activities*, *Stress Management and Spiritual Participation*, and *Leisure*) showed good score agreement: 0.92 (0.83–0.96), 0.86 (0.72–0.93), 0.79 (0.59–0.90), and 0.75 (0.53–0.88), respectively. Three (*Activities of Daily Living*, *Diet*, and *Other Health Promotion and Risk Behaviors*) showed moderate score agreement: 0.71 (0.46–0.85), 0.64 (0.35–0.81), and 0.57 (0.26–0.77), respectively (Table 3). Most items in the *HELP-T* showed moderate-to-good individual item agreement.

### Validity

Most items reached an acceptable level of validity and were significantly correlated (*p* < 0.05) (Table 3). The construct validity of the *HELP-T* was supported by the significant correlation between the individual subscales and the total score (Spearman *rho*_s_ = 0.41–0.67, *p* < 0.01) (Table 4). The significant small-to-moderate positive correlations between most of the subscale pairs support our hypothesis. However, the *Diet* subscale was not significantly correlated with four other subscales (*Exercise*, *Social and Productive Activities*, *Leisure*, and *Stress Management and Spiritual Participation*).

**Table 4:** Interrelationships (Spearman’s *rho*) between the *HELP-T* subscales.

Domains	Exercise	Diet	Social^†^	Leisure	ADLs^‡^	Stress management^§^	Health behavior^¶^	Total
Exercise	1	0.10	0.32**	0.42**	0.09	0.17**	0.35**	0.57**
Diet	-	1	-0.01	0.11	0.21**	0.11	0.26**	0.41**
Social^†^	-	-	1	0.36**	0.13*	0.32**	0.29**	0.57**
Leisure	-	-	-	1	0.19**	0.36**	0.42**	0.66**
ADLs^‡^	-	-	-	-	1	0.20**	0.18**	0.45**
Stress management^§^	-	-	-	-	-	1	0.32**	0.62**
Health behavior^¶^	-	-	-	-	-	-	1	0.67**
**Total**	-	-	-	-	-	-	-	1

*: *p* < 0.05

**: *p* < 0.01. Spearman’s *rho* was 0.41–0.67 between each subscale and the total, and it is acceptable.

^†^: Social: *Social and productive activities*

^‡:^ ADLs: *Activities of daily living*

^§:^ Stress management: *Stress management and spiritual participation*

^¶:^ Health behavior: *Other health promotion and risk behaviors*.

Mann-Whitney U tests showed significant differences (*p* < 0.0001) in the total score and in all subscale scores (except *Stress Management and Spiritual Participation*) of participants who rated their health as excellent or good and of those who rated their health as fair or poor (Table 5). These findings supported the discriminant validity of the *HELP-T*: the *HELP-T* distinguished between older adults with different self-rated levels of health.

**Table 5:** Comparisons between participants who rated their health as excellent or good and those who rated their health as fair or poor.

Domain	Good-to-excellent	Poor-to-fair	*p*
	health (n = 84)	health (n = 187)	
Exercise	5.57 (3.48)	4.35 (2.82)	0.007**
Diet	12.05 (2.85)	11.27 (2.53)	0.008**
Social and productive activities	4.60 (3.12)	3.76 (2.89)	0.026*
Leisure	5.61 (2.40)	4.98 (2.57)	0.020*
Activities of daily living	12.20 (2.24)	11.35 (2.51)	0.013*
Stress management and	6.37 (3.28)	5.81 (3.18)	0.221
spiritual participation			
Other health promotion and risk			
behaviors	10.88 (2.57)	10.08 (2.22)	0.015*
**Total**	**57.20 (12.06)**	**51.73 (10.37)**	0.001**

Means between the two groups were compared using a Mann-Whitney U test.

*: *p* < 0.05

**: *p* < 0.01.

## Discussion

The 59-item *HELP-T* is the first health-related lifestyle assessment designed specifically for older Taiwanese adults. The *HELP-T* was culturally adapted from the original 56-item English version by adding two culturally specific items about family activities and by splitting one *Leisure* item into two. The original 6-point rating scale was modified to a 3-point scale to make it easier for older Taiwanese adults to use. The *HELP-T* total score had good internal consistency, and most *HELP-T* domains showed acceptable-to-good test-retest reliability and good construct validitythe *HELP-T* is suitable for measuring various aspects of lifestyle factors and behaviors in older Taiwanese adults.

Health is generally conceptualized in some dimensions that are universal across cultures, but other dimensions vary by culture [22–24]. We added two items about family activities because they are central to Chinese culture. A recent *National Survey* [25] reported that 82.8% of older Taiwanese adults get together with their children at least once a week. Our study confirmed that health-related lifestyle activity profiles completed by older Taiwanese adults reflect their social and cultural values.

Although the original 6-point rating scale might make the *HELP* more sensitive by reflecting small incremental lifestyle changes, we used a 3-point scale in the *HELP-T* for three reasons. First, our statistical analysis indicated that the scores from the two rating scales were highly correlated. Second, too many choices can compromise a person’s decisiveness, especially for the elderly and those with a low level of formal education [26]. Compared with the original *HELP* study [15], in which 91.2% of the participants in the U.S. had completed secondary or higher education, only 66.1% of our participants had. Third, because of potential variations in the lifestyle context within the 3-month survey time-frame (e.g., holidays and other special events), some participants commented that they could recall only the approximate frequency for each of the activities and behaviors included in the *HELP-T* and that 6 choices made the questions difficult to answer.

The internal consistency of the *HELP-T* was acceptable. The test-retest reliability was good overall, and it was fair-to-good for all subscales, but “*Diet*” and “*Other Health Promotion and Risk Behaviors*”, for which it was unsatisfactory. We found that many items in these subscales depended upon changes in social and temporal contexts. For example, within a week, one may have several social events involving eating too much or eating unhealthy food and drinking too much alcohol (e.g., banquets, parties, rituals). Similarly, it is common for older Taiwanese adults to take over-the-counter medications for mild and brief symptoms of illness (e.g., pain, cold). These behaviors might have jeopardized our test-retest results.

The construct validity of the *HELP-T* was first supported by the interrelationships between the different lifestyle behaviors subscales. The significant low-to-moderate correlations between the 7 subscales indicate that each subscale contributes a somewhat related but distinctive aspect to the measure of a healthy lifestyle. Clinically, the scores from different *HELP-T* subscales can help identify areas of strength and weakness in a person’s lifestyle. Thus, service planning can be individualized to meet each older adult’s personal needs.

The construct (discriminant) validity of the *HELP-T* was also supported by the ability of the scores to distinguish between participants who perceived themselves to be in good health and those who did not. Lifestyle behaviors are reported [27] to be responsible for at least 50% of how healthy one is. Others have reported that older adults who perceive their health as poor are less likely to exercise [28] and perform self-care [29], and that they are more likely to engage in risky behaviors, such as smoking, heavy alcohol drinking, and poor eating habits [30]. Our results echo these previous findings [16].

It is noteworthy that, although the original *HELP* and the *HELP-T* consist of 7 subscales that yield subtotal scores, a healthy lifestyle does not entail a high score for every subscale. Individuals must prioritize their own needs to develop personal plans that allow them to achieve a balanced, healthy lifestyle. We recently also developed a *HELP-T* Intervention Plan Form along with a Clinician Guide [31], in which an individual client and the clinician are instructed to establish their goals for change and to identify their targeted *HELP-T* activities to achieve the goals.

The generalizability of our findings is limited, however, because we enrolled only a small nonrandom sample from Taiwan. Future studies should include larger and more representative random samples of older adults in Taiwan.

## Conclusions

This study adapted the *HELP* for cross-cultural use with older Taiwanese adults. We modified both the content and the rating scale to make *HELP-T* suitable for older Taiwanese adults. The *HELP-T* is a valid and useful tool that enables clinicians to understand the health-promoting habits and routines of older Taiwanese adults, helps them establish goals for lifestyle change, and yields client-centered lifestyle monitoring and recommendations.

## Supporting information

S1 File Appendix. Coded book of demographic data and *HELP-T* results of all participants.
(XLSX)
